# Machine learning-based prediction of acute mortality in emergency department patients using twelve-lead electrocardiogram

**DOI:** 10.3389/fcvm.2023.1245614

**Published:** 2023-10-27

**Authors:** Po-Cheng Chang, Zhi-Yong Liu, Yu-Chang Huang, Yu-Chun Hsu, Jung-Sheng Chen, Ching-Heng Lin, Richard Tsai, Chung-Chuan Chou, Ming-Shien Wen, Hung-Ta Wo, Wen-Chen Lee, Hao-Tien Liu, Chun-Chieh Wang, Chang-Fu Kuo

**Affiliations:** ^1^ Division of Cardiology, Department of Internal Medicine, Chang Gung Memorial Hospital, Linkou and Chang Gung University Medical School, Taoyuan, Taiwan; ^2^ Center for Artificial Intelligence in Medicine, Chang Gung Memorial Hospital, Taoyuan, Taiwan; ^3^ School of Biomedical Informatics, University of Texas Health Science Center at Houston, Houston, TX, United States; ^4^ Division of Rheumatology, Allergy and Clinical Immunology, Chang Gung Memorial Hospital, Linkou and Chang Gung University Medical School, Taoyuan, Taiwan

**Keywords:** mortality, emergency department, convolutional neural network, machine learning, electrocardiogram

## Abstract

**Background:**

The risk of mortality is relatively high among patients who visit the emergency department (ED), and stratifying patients at high risk can help improve medical care. This study aimed to create a machine-learning model that utilizes the standard 12-lead ECG to forecast acute mortality risk in ED patients.

**Methods:**

The database included patients who visited the EDs and underwent standard 12-lead ECG between October 2007 and December 2017. A convolutional neural network (CNN) ECG model was developed to classify survival and mortality using 12-lead ECG tracings acquired from 345,593 ED patients. For machine learning model development, the patients were randomly divided into training, validation and testing datasets. The performance of the mortality risk prediction in this model was evaluated for various causes of death.

**Results:**

Patients who visited the ED and underwent one or more ECG examinations experienced a high incidence of 30-day mortality [18,734 (5.42%)]. The developed CNN model demonstrated high accuracy in predicting acute mortality (hazard ratio 8.50, 95% confidence interval 8.20–8.80) with areas under the receiver operating characteristic (ROC) curve of 0.84 for the 30-day mortality risk prediction models. This CNN model also demonstrated good performance in predicting one-year mortality (hazard ratio 3.34, 95% confidence interval 3.30–3.39). This model exhibited good predictive performance for 30-day mortality not only for cardiovascular diseases but also across various diseases.

**Conclusions:**

The machine learning-based ECG model utilizing CNN screens the risks for 30-day mortality. This model can complement traditional early warning scoring indexes as a useful screening tool for mortality prediction.

## 1. Introduction

Patients admitted to the emergency department (ED) have a considerable risk of mortality, estimated to be between 3% and 8% for 30-day mortality (1, 2). Identifying high-risk patients early on can help make appropriate medical management decisions. Early warning scores (EWS) based on simple and widely available parameters are valuable tools for predicting acute mortality risk. Various EWS, including the National Early Warning Score (NEWS) (3), Modified Early Warning Score (MEWS) (4), Rapid Acute Physiology Score (RAPS) (5), Rapid Emergency Medicine Score (REMS) (6), and Cardiac Arrest Risk Triage Score (CART) (7), have been developed to assess acute mortality risk. Immediate risk stratification guides medical staff in making appropriate emergent management decisions and arranging admission to the intensive care unit.

The 12-lead electrocardiogram (ECG) is an important medical test in the ED, and most high-risk patients who present to the ED undergo on or more ECG examinations. Physicians diagnose various medical disorders, such as cardiovascular diseases, arrhythmias, and electrolyte disorders via reading the ECG. Alterations of medical conditions can cause ECG changes, some of which are easily recognized, while others are subtle and difficult to interpret visually by physicians. It can be challenging for physicians to assess the mortality risk using a 12-lead ECG examination. However, the use of convolutional neural network (CNN) machine learning allows for the recognition of these subtle ECG changes.

Detecting a high risk of acute mortality early on with a 12-lead ECG examination can aid in risk stratification for ED patients. In this study, we aimed to develop a CNN machine learning model using the standard 12-lead ECG to predict acute mortality risk in patients who visit the ED.

## 2. Methods and materials

### 2.1. Study population

This study was approved by the Institutional Review Board (IRB No. 202002464B0). The database of this study included all patients who visited the emergency departments and underwent standard 12-lead ECG at the seven hospitals between October 2007 and December 2017. Patients who visited the EDs and received one or more standard 12-lead ECG examinations during their visit were included in this study. The demographics, medical history, medications, and laboratory data were acquired from the Chang Gung Research Database. The survival status was acquired from the National Death Registry Database of Taiwan. All the data were de-identified before analyses, and all personal information was encrypted before the data were released to researchers to protect patient confidentiality. Since the NEWS, MEWS, RAPS, REMS, and CART indexes are derived from the Glasgow coma scale (GCS), oxygen saturation, body temperature, pulse rate, blood pressure, and respiratory rate. We excluded subjects missing any of the EWS index data in the database.

### 2.2. ECG collection and artificial intelligence (AI) model development

Standard 12-lead ECGs with 10-second voltage-time traces were acquired using MAC 5,000 or MAC 5,500 ECG recorder (GE Healthcare, Chicago, IL, USA) at a sampling rate of 500 Hz. After ECG acquisition, the ECG tracings were processed and stored using the Marquette Universal System for Electrocardiography (MUSE, GE Healthcare, Chicago, IL, USA). If a patient has two or more ECG records, we used all ECGs during their ED visit. Each standard 12-lead ECG was stored as a 5,000 × 12 matrix.

For signal input, we used the convolutional network framework (CNN) residual network (ResNet 18) (8) but modified it to fit our signal input (Supplementary Figure S1). We used a wider kernel 15 for the first convolution layer compared with the original ResNet framework as used for the image. This architecture uses skip connections, which allow information to directly pass to the next layer to avoid the degradation caused by deeper neural networks. The network consisted of a convolution layer followed by 4 residual blocks, and each residual block contains two convolution layers. The output of the last block was fed into hybrid pooling (9) by combining max- and average-pooling methods to improve the generalization ability while reducing dimensionality. The output of hybrid pooling was later sent to a fully connected layer to perform the final classification. The output of each convolutional layer is followed by batch normalization for distribution normalization and fed into a rectified linear activation unit (10). Cross-entropy loss with Adam (11) optimizer was used in the model. Dropout is applied to reduce overfitting by breakup co-adaptation on training data (12).

The AI ECG model incorporated ECG and the mortality scoring systems were analyzed based on the abovementioned model (Supplementary Figure S2). The additional scoring system variables were sent to a fully connected layer and combined max- and average-pooling of the ECG model. The output was later sent to a fully connected layer to perform the final classification.

### 2.3. Statistical analyses

The Kolmogorov-Smirnov test was utilized to assess normality due to the substantial sample size exceeding 2,000. Consequently, all *P*-values were less than 0.05, leading to the rejection of the assumption of normality. Continuous variables are expressed as median and interquartile range (IQR), and categorical variables are expressed as numbers and percentages. Adjusted odds ratios (OR) and 95% confidence interval (CI) were calculated. For comparisons of population characteristics, the chi-square test was used for categorical variables and the unpaired Student's *t*-test for continuous variables. Cox proportional hazards were used to estimate hazard ratios (HR) for mortality. A *P*-value < 0.05 was considered statistically significant. Statistical analyses were conducted using SAS 9.4 software (SAS Institute, Cary, NC, USA).

## 3. Results

### 3.1. Clinical characteristics

**Figure 1:**
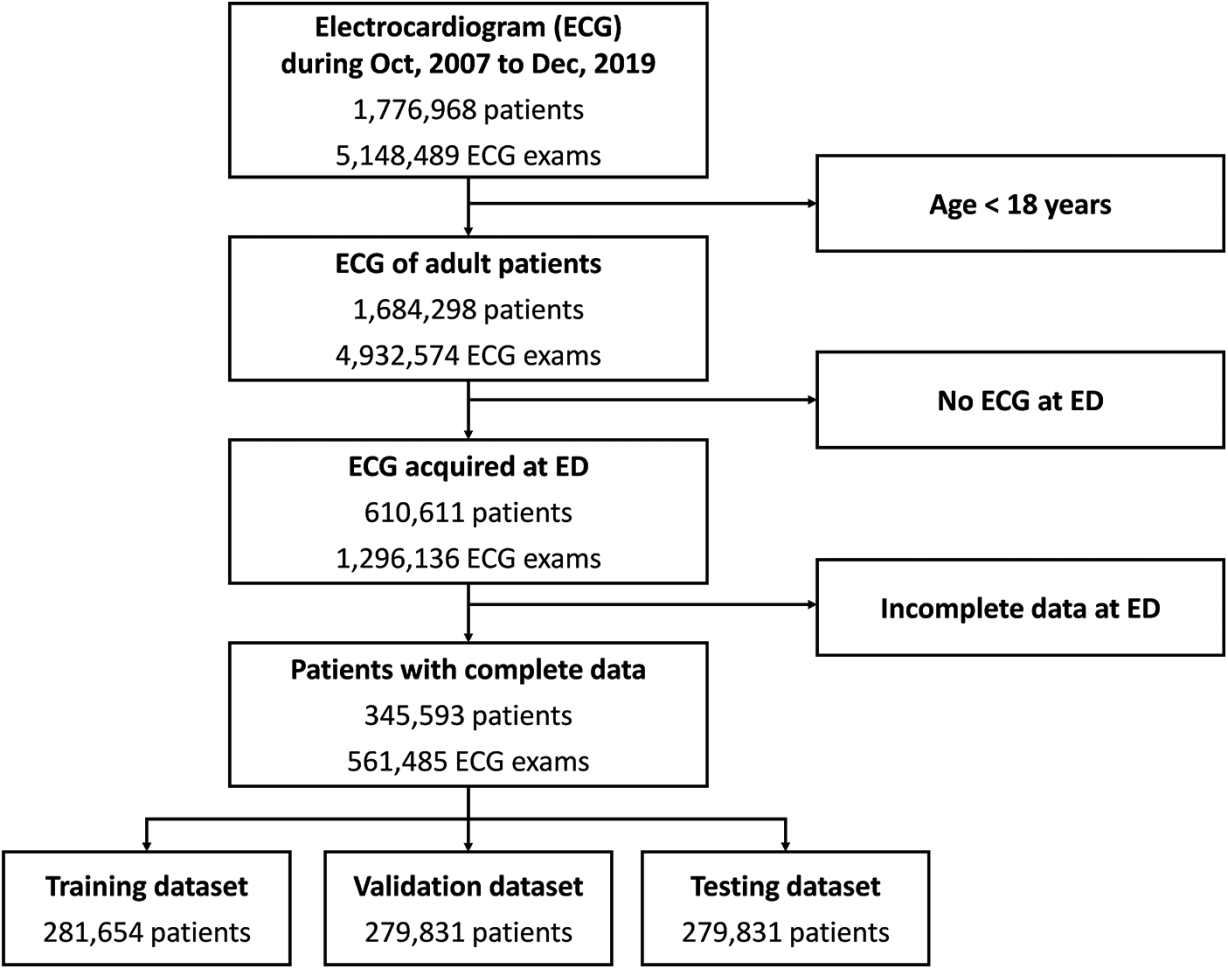
Data flow for ECG and data pairing. Patients who visited the emergency departments between 2006 and 2017 were included in this study. ECG, electrocardiogram; ED, emergency department.

The dataset comprised 5,148,498 standard 12-lead ECG examinations from 1,776,968 patients collected between October 2007 and December 2019 (Figure 1). Among these patients, 1,684,298 had recorded data in the National Health Insurance or National Death Registry Databases, from which we obtained the mortality outcome and the primary cause of death. After excluding patients with inadequate ECG quality and those age less than 18 years, a total of 610,611 patients were included. We excluded 265,018 patients due to incomplete ED triage data, and the remaining 345,593 patients were randomly divided into training and testing datasets. Table 1 shows the clinical characteristics.

**Table 1:** Clinical patient characteristics.

	All (*N *= 561,485)	Training (*N* = 196,990)	Validation (*N* = 84,664)	Testing (*N* = 279,831)
Demographics				
Age (years)	65.3 (26.3)	65.2 (26.2)	65.5 (26.2)	65.3 (26.3)
Female	260,465 (46.4)	91,579 (46.5)	39,142 (46.2)	129,744 (46.4)
Medical history				
Hypertension	292,430 (52.1)	102,626 (52.1)	44,213 (52.2)	145,591 (52.0)
Diabetes	171,872 (30.6)	59,725 (30.3)	26,101 (30.8)	86,046 (30.7)
Hyperlipidemia	153,758 (27.4)	53,861 (27.3)	23,785 (28.1)	76,112 (27.2)
Old MI	45,818 (8.2)	16,083 (8.2)	6,875 (8.1)	22,860 (8.2)
Heart failure	98,578 (17.6)	34,735 (17.6)	15,008 (17.7)	48,835 (17.5)
Atrial fibrillation	60,284 (10.7)	20,870 (10.6)	9,316 (11.0)	30,098 (10.8)
Chronic kidney disease	91,674 (16.3)	32,444 (16.5)	14,039 (16.6)	45,191 (16.1)
Liver cirrhosis	33,730 (6.0)	11,792 (6.0)	5,003 (5.9)	16,935 (6.1)
COPD	129,948 (23.1)	45,458 (23.1)	20,036 (23.7)	64,454 (23.0)
Previous medications				
ACEis or ARBs	295,064 (52.6)	103,653 (52.6)	44,742 (52.8)	146,669 (52.4)
Beta-blockers	218,319 (38.9)	76,398 (38.8)	33,204 (39.2)	108,717 (38.9)
Calcium channel blockers	271,419 (48.3)	95,235 (48.3)	40,967 (48.4)	135,217 (48.3)
Statins	182,849 (32.6)	63,780 (32.4)	28,007 (33.1)	91,062 (32.5)
Lab data				
Hemoglobin (g/dl)	13.6 (2.3)	13.7 (2.3)	13.6 (2.3)	13.6 (2.2)
WBC (/mm^3^)	8.4 (4.9)	8.4 (4.9)	8.4 (4.8)	8.4 (4.9)
Neutrophils (/mm^3^)	72.3 (19.8)	72.3 (19.7)	72.2 (19.8)	72.4 (19.8)
Lymphocytes (/mm^3^)	18.7 (17.0)	18.6 (16.9)	18.7 (17.0)	18.6 (17.1)
Creatinine (mg/dl)	0.8 (0.3)	0.8 (0.3)	0.8 (0.3)	0.8 (0.3)
BUN (mg/dl)	18.2 (18.4)	18.2 (18.5)	18.2 (18.4)	18.2 (18.4)
ALT (unit/L)	21.0 (18.0)	21.0 (18.0)	21.0 (18.0)	21.0 (18.0)
Troponin-I (ng/ml)	0.016 (0.025)	0.017 (0.025)	0.017 (0.025)	0.016 (0.025)
CRP (mg/L)	15.0 (56.4)	15.1 (57.2)	14.9 (55.2)	14.9 (56.3)

Continuous variables are presented as median (interquartile range); categorical variables are presented as number (percentage).

ACEi, angiotension-converting enzyme inhibitors; ALT, alanine aminotransferase; ARB, angiotensinogen receptor blockers; BUN, blood urea nitrogen; COPD, chronic obstructive pulmonary disease; CRP, C-reactive protein; MI, myocardial infarction; WBC, white blood cell.

### 3.2. Acute mortality prediction outcomes

**Figure 2:**
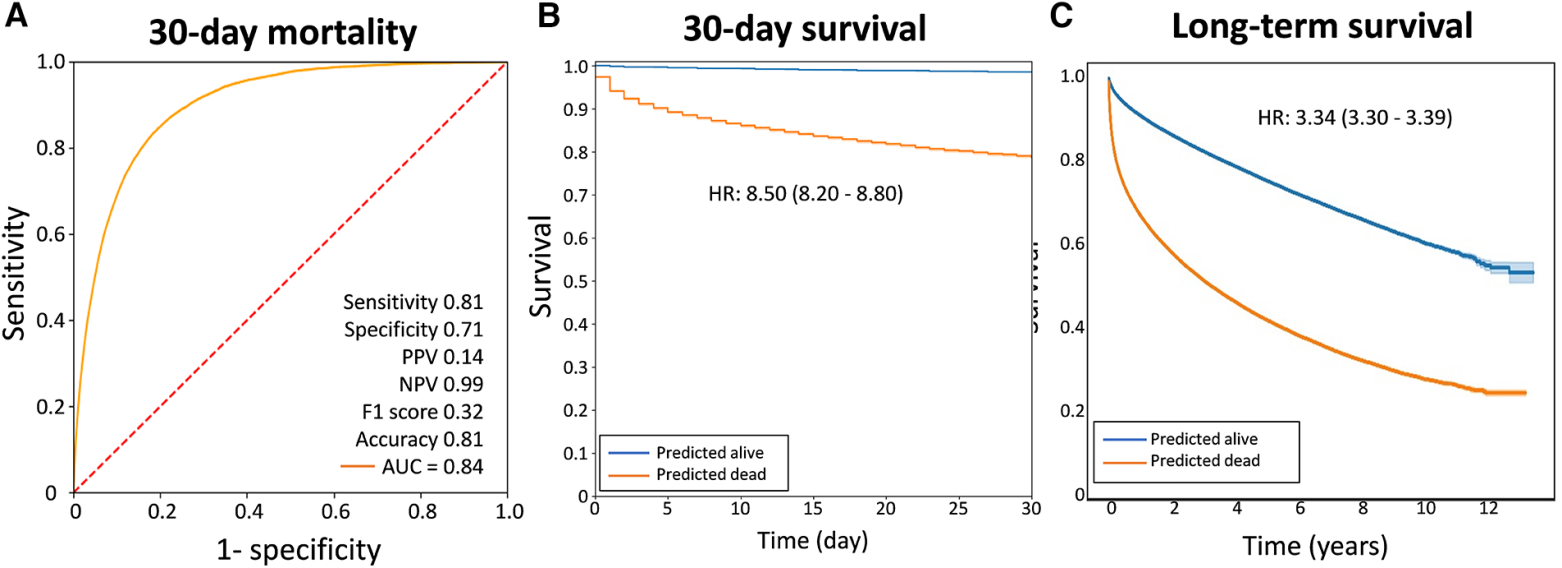
The performance of the AI ECG model in predicting acute mortality. (**A**) The receiver operating characteristic curves. (**B**) The Kaplan–Meier curves of 30-day survival. (**C**) The Kaplan–Meier curves of extenteded survival prediction using the same model. The graphs demonstrated that this model performed well in predicting one-year mortality. AUC, area under the receiver operating characteristic curve; HR, hazard ratio; NPV, negative predictive value; PPV, positive predictive value.

Among these patients, 18,734 (5.42%) died within 30 days, indicating a relatively high 30-day mortality risk among ED patients who received one or more ECG examinations. The CNN model showed a good performance in predicting 30-day mortality. The sensitivity, specificity, and negative predictive values were 0.81, 0.71, and 0.99, respectively. Patients who predicted that they would die had a 19% risk of mortality within 30 days, whereas patients who predicted that they would survive had a 1% risk of mortality within 30 days. Figure 2A shows the ROC curve of acute mortality prediction, with the area under the ROC curve of 0.84. Figure 2B demonstrates the Kaplan–Meier curve of 30-day mortality (odds ratios 8.50, 95% CI 8.20–8.80). Although the CNN model was originally developed for short-term mortality, it demonstrated good performance in predicting long-term mortality as well (Figure 2C). The group predicted to be at high mortality risk had a significantly higher one-year mortality rate than the group predicted to be alive in this mortality prediction model (hazard ratio 3.34, 95% CI 3.30–3.39).

**Figure 3:**
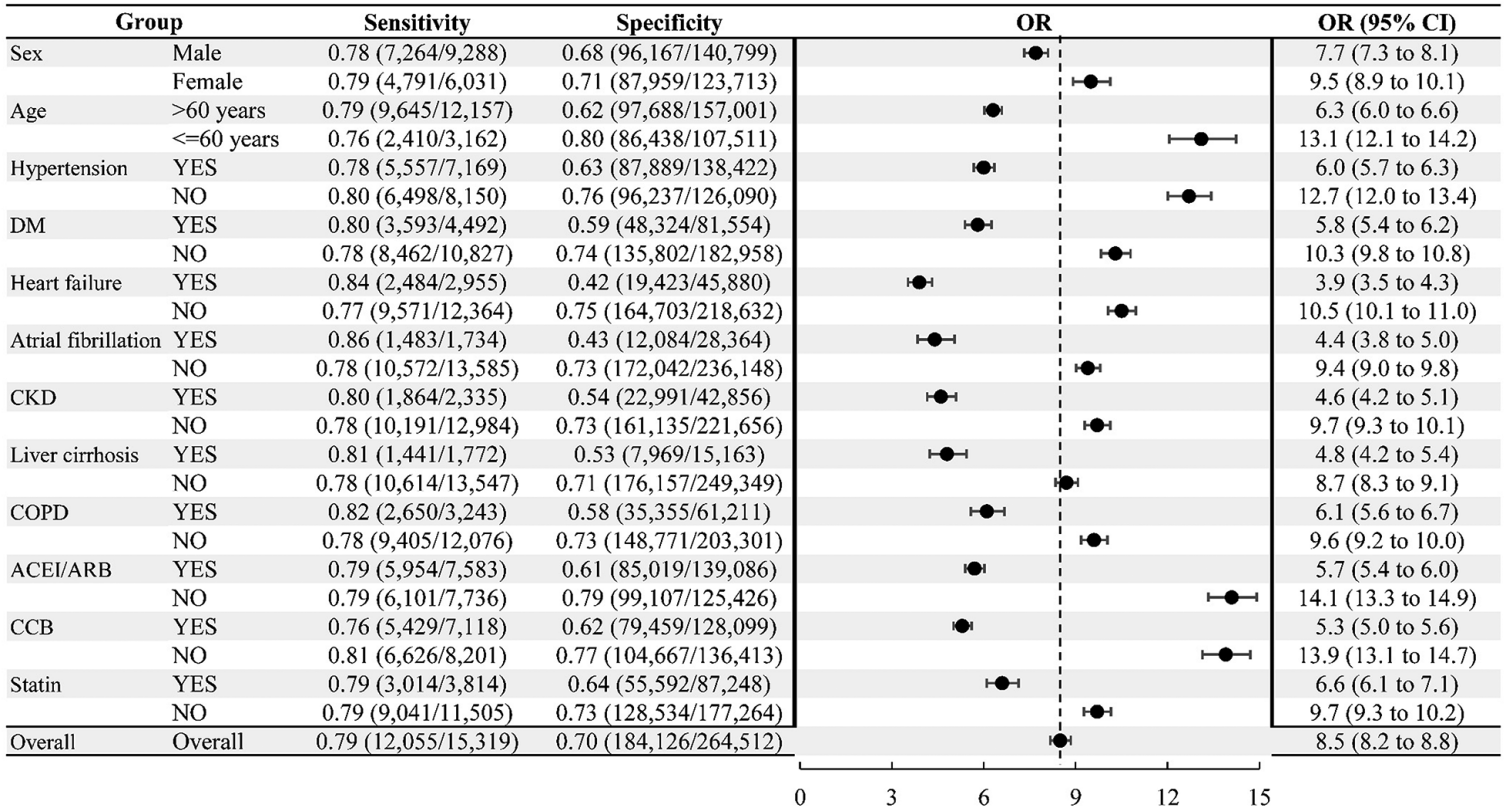
Subgroup analyses for 30-day emergency department mortality. ACEI/ARB, angiotensin-converting enzyme inhibitor/angiotensin receptor blocker; CCB, calcium channel blocker; CI, confidence interval; CKD, chronic kidney disease; COPD, chronic obstructive pulmonary disease; DM, diabetes mellitus; OR, odds ratio.

**Figure 4:**
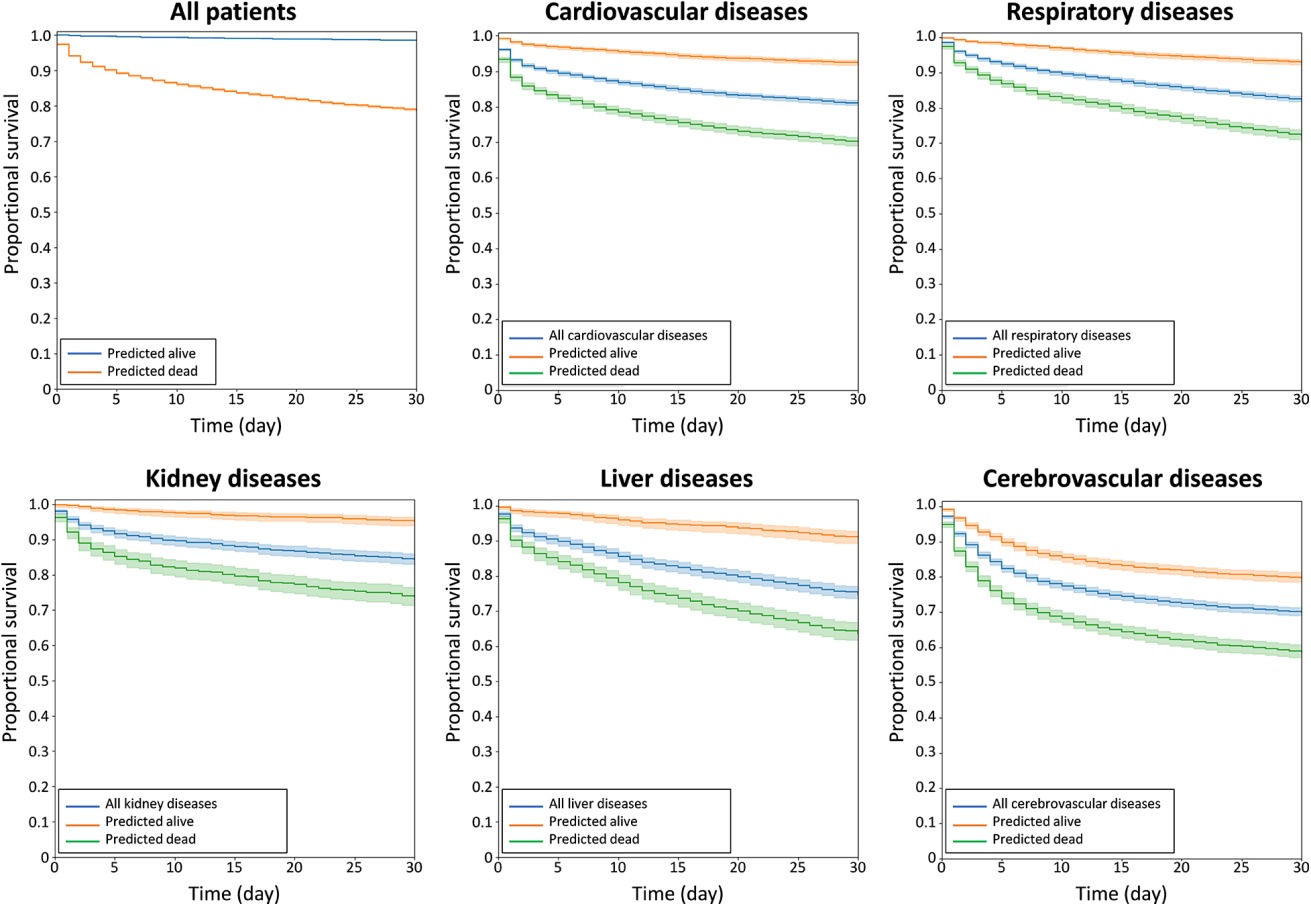
Kaplan–MMeier curves of acute survival prediction in patients among various major diseases, including cardiovascular, respiratory, kidney, liver, and cerebrovascular diseases.

The model has acute mortality prediction ability in all subgroups, including older patients (>60 years old), hypertension, diabetes, heart failure, atrial fibrillation, chronic kidney disease, liver cirrhosis, chronic obstructive lung disease, and those taking angiotensin-converting enzyme inhibitors/angiotensin II receptor blockers, calcium channel blockers and statins as revealed by the subgroup analyses (Figure 3). The model exhibited good predictive performance for 30-day mortality across a range of diseases, as illustrated in Figure 4. This included cardiovascular, respiratory, kidney, liver, cerebrovascular, and malignancy diseases.

Since we employed a multimodal machine learning approach, we also analyzed the performances of machine learning using ECG and the EWS index (see Supplementary Table S1). Our findings indicate that multimodal machine learning, incorporating both ECG and EWS indexes, performed better than machine learning using ECG data alone. Furthermore, the performance of machine learning using only the EWS indexes was the poorest.

## 4. Discussions

In this study, machine learning models were developed to predict 30-day mortality in patients admitted to the ED. The models showed good performance in predicting 30-day mortality. The Kaplan–Meier curve demonstrates that the model performed well for predicting 30-day survival as well as for predicting long-term survival. The performance was good across all subgroups, as demonstrated by subgroup analyses. The ECG model also performed well in predicting mortality across various diseases, including cardiovascular, respiratory, kidney, liver, and cerebraovascular diseases.

### 4.1. Acute mortality prediction for patients admitted to the emergency department

High-risk patients with cardiovascular, respiratory, kidney, and liver diseases may require early intensive monitoring and care. EWS indexes, including MEWS, NEWS, and qSOFA, are widely used in the ED to stratify risk for early intensive health care. The NEWS index is a commonly used prediction model for early detection of clinical deterioration, which is based on vital signs and consciousness levels, making it a simple and straightforward scoring system (13). Compared to the MEWS and the qSOFA scoring system, NEWS is one of the most accurate tools for predicting mortality within 24 h (14). In previous reports (14–17), the area under the ROC curve for predicting short-term (7–30 days) mortality in patients who visited ED was 0.61–0.81.

Predicting acute mortality can aid clinicians in managing patients at the appropriate time. The EWS indexes use readily available clinical data, including vital signs, oxygen saturation, and consciousness levels. However, the ECG data might be altered by cardiovascular disease, electrolytes, autonomic activities, intracranial diseases, and other systemic diseases. Subtle changes associated the systemic diseases can be identified using the ECG CNN model for predicting acute mortality. The ECG model complements EWS indexes and can effectively predict the risk of acute mortality, enabling clinicians to make early decisions in critical medical care. As the 12-lead ECG is one of the most readily available examinations in the emergency department, combining an ECG examination with the scoring systems could aid in risk stratification and reduce waiting times for intensive care.

### 4.2. The performance of acute vs. one-year mortality prediction

Initially, we trained a model to predict one-year mortality and evaluated its ability to predict mortality on a monthly basis. The ECG machine learning model exhibited superior predictive accuracy for patients with a high risk of mortality within a month (refer to Supplementary Figure S2). The model's monthly accuracy indicated that it performed best during the first month and gradually declined after the first month. The model's superior performance in predicting acute mortality during the first month indicates that it is better suited for predicting acute mortality. This better performance during the first month may be attributed to ECG changes that reflect the immediate systemic clinical condition. In addition, the most critical concern for patients who seek emergency medical attention is the prediction of acute mortality, rather than one-year mortality. Thus, we modified the model to predict acute mortality. However, the acute-mortality model still performs effectively in predicting long-term mortality.

### 4.3. The future application of mortality prediction in preventive medicine

Cardiovascular diseases are the primary cause of death in developed countries, with atherosclerotic coronary heart disease (CHD) having major risk factors that include diabetes, hypertension, increased total serum cholesterol, high LDL level, low HDL level, cigarette smoking, obesity, and family history of CHD. Accurate prediction of CHD events is crucial for guiding decisions on preventive therapy for hypertension, diabetes, and dyslipidemia. The Pooled Cohort Equation is a commonly used method to estimate 10-year absolute rates of CHD events in a primary prevention population (18). However, most CHD prediction models are based on major risk factors (19–22) and provide no information on overall survival, although they do offer valuable risk information for clinicians to decide whether to prescribe preventive therapy. This ECG model exhibited good prediction performance for most major causes of death, suggesting that the ECG signals are influenced by not only cardiovascular diseases but also other systemic diseases. Disease progression may cause changes in the ECG, some of which may be subtle, but recognizable by the CNN ECG model. Therefore, the CNN ECG model may help clinicians evaluate the future risk of acute as well as long-term mortality for various diseases.

For clinical ED staff, risk/mortality screening tools, such as early warning indexes, can be helpful in identifying patients at a relatively higher risk. For patients with a lower risk, the staff can continue to observe them until they exhibit high-risk warning signs. Clinical staff can allocate more attention to the high-risk group. Therefore, a clinical screening tool with low PPV is acceptable.

### 4.4. Limitations

Some limitations exist in this study. Firstly, only ECGs recorded using GE Healthcare ECG recorders were analyzed, so the model's performance may be poorer for ECGs recorded with other recorders. As the model is based on a convolutional neural network, the algorithm cannot provide complete interpretability of mortality. Additionally, the majority of ECGs analyzed in this study were obtained from Asian patients, and hence the generalizability of the model for mortality prediction may be restricted. Patients in the ED who undergo one or more ECG exams typically have more comorbidities and a higher risk of mortality. The ECG-based machine learning model may not be effective for all ED patients. Moreover, the machine learning study is based on a convolutional neural network, and the interpretability of mortality prediction is currently limited.

## 5. Conclusions

The machine learning-driven ECG model is a reliable screening tool that provides reasonably accurate predictions for 30-day mortality. The study confirms that this model performs well not only for cardiovascular diseases but also for other medical disorders. The machine learning model's ability to swiftly predict acute mortality based on twelve-lead ECGs can assist clinicians in managing patients who visit the ED. Furthermore, the machine learning-driven ECG model can complement traditional EWS as a useful screening tool.

## Data Availability

The original contributions presented in the study are included in the article/**Supplementary Material**, further inquiries can be directed to the corresponding authors.
