# Supplementary material for: Machine learning-based prediction of acute mortality in emergency department patients using twelve-lead electrocardiogram
Source: Front Cardiovasc Med. 2023 Oct 27;10:1245614. doi: 10.3389/fcvm.2023.1245614 (PMC10641780; doi:10.3389/fcvm.2023.1245614)
Supplement: Supplementary file 1 [file Presentation1.pdf]

Supplementary Table. Comparisons of the ECG AI model and major early warning score (EWS) indexes

Models	3-day mortality					7-day mortality					30-day mortality				
	AUC	Sensitivity	Specificity	PPV	NPV	AUC	Sensitivity	Specificity	PPV	NPV	AUC	Sensitivity	Specificity	PPV	NPV
NEWS	0.85	0.004	1.00	0.35	0.98	0.83	0.01	1.00	0.37	0.98	0.79	0.03	1.00	0.42	0.95
MEWS	0.82	0.01	1.00	0.41	0.98	0.80	0.01	1.00	0.48	0.98	0.76	0.02	1.00	0.38	0.95
RAPS	0.69	0.00	1.00	1.00	0.98	0.66	0.00	1.00	1.00	0.98	0.60	0.00	1.00	0.00	0.95
REMS	0.74	0.00	1.00	1.00	0.98	0.73	0.00	1.00	0.20	0.98	0.69	0.002	1.00	0.36	0.95
CART	0.76	0.00	1.00	0.40	0.98	0.75	0.00	1.00	0.40	0.98	0.74	0.01	1.00	0.31	0.95
Autoscore	0.81	0.73	0.75	0.04	0.99	0.80	0.71	0.75	0.07	0.99	0.82	0.74	0.74	0.14	0.98
AI ECG + NEWS	0.83	0.79	0.72	0.04	0.99	0.82	0.81	0.69	0.06	0.99	0.81	0.79	0.70	0.13	0.98
AI ECG + MEWS	0.88	0.82	0.80	0.06	0.99	0.88	0.83	0.78	0.09	0.99	0.86	0.81	0.75	0.16	0.99
AI ECG + RAPS	0.88	0.83	0.77	0.05	0.99	0.88	0.82	0.78	0.09	0.99	0.86	0.79	0.77	0.16	0.98
AI ECG + REMS	0.88	0.83	0.78	0.06	0.99	0.88	0.84	0.77	0.08	0.99	0.85	0.80	0.75	0.16	0.99
AI ECG + CART	0.88	0.81	0.80	0.06	0.99	0.88	0.81	0.81	0.09	0.99	0.86	0.82	0.75	0.16	0.99
AI ECG	0.85	0.79	0.74	0.05	0.99	0.85	0.79	0.75	0.07	0.99	0.84	0.81	0.71	0.14	0.99
AI ECG + all indexes	0.92	0.87	0.82	0.09	1.00	0.91	0.86	0.81	0.12	0.99	0.90	0.86	0.79	0.19	0.99

AI, artificial intelligence; AUC, area under receiver operating characteristic curve; CART, Cardiac Risk Assessment Triage; ECG, electrocardiogram; MEWS, Modified Early Warning Score; NEWS, National Early Warning Score; NPV, negative predictive value; PPV, positive predictive value; RAPS, Rapid Acute Physiology Score; REMS, Rapid Emergency Medicine Score.
