# Supplementary material for: Machine learning-based prediction of acute mortality in emergency department patients using twelve-lead electrocardiogram
Source: Front Cardiovasc Med. 2023 Oct 27;10:1245614. doi: 10.3389/fcvm.2023.1245614 (PMC10641780; doi:10.3389/fcvm.2023.1245614)

Supplementary Figure 1

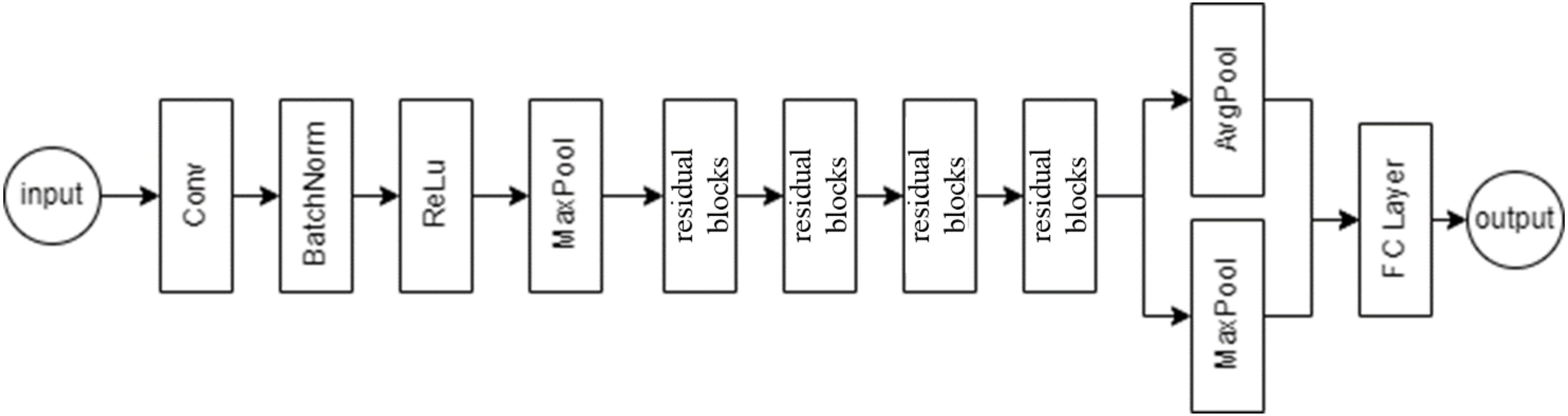

Supp Fig 1. The modified convolutional network framework data flow of this electrocardiogram artificial intelligence model.

Supplementary Figure 2

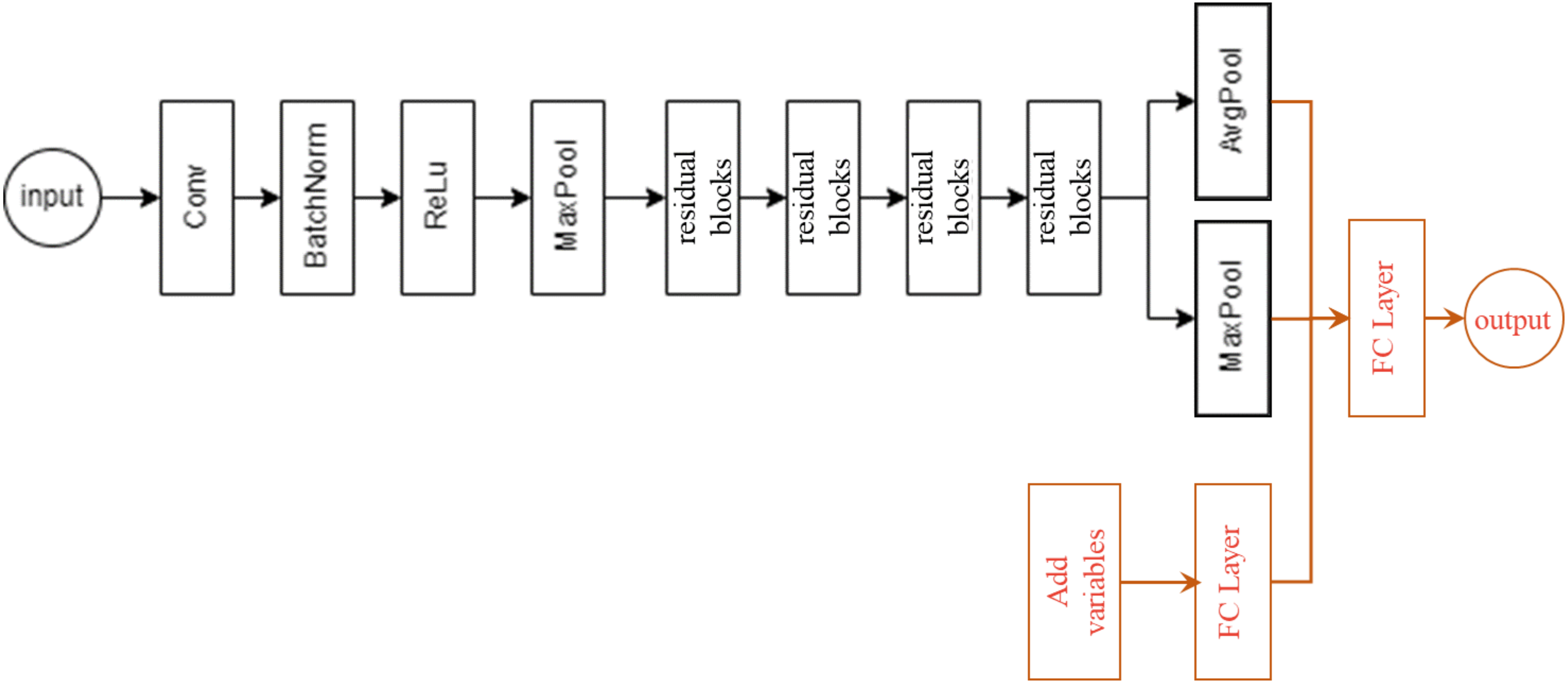

Supp Fig 2. The convolutional network framework data flow incorporated electrocardiogram and the mortality scoring systems based on the abovementioned model.

Supplementary Figure 2

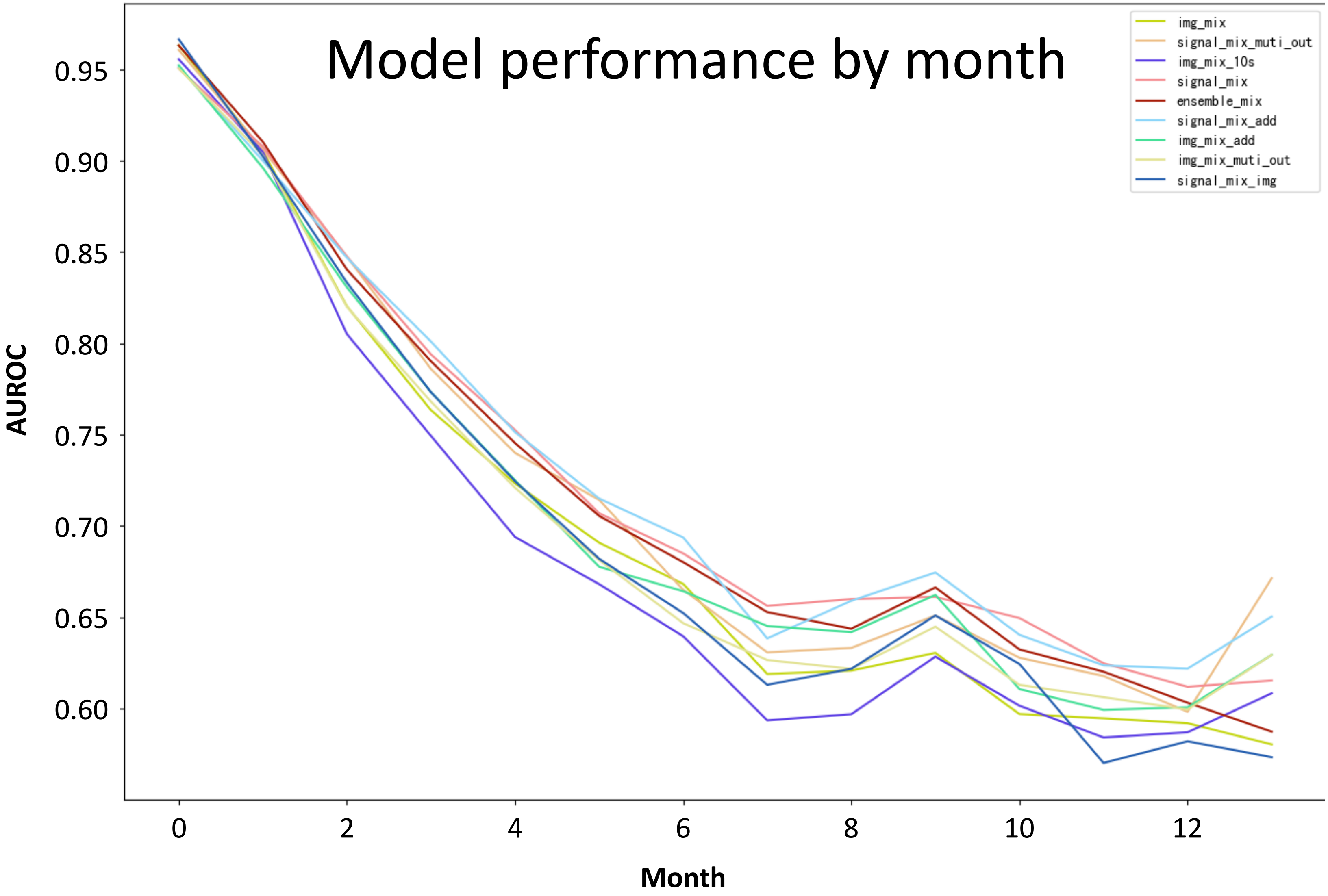

Supplement: Supplementary file 2 [file Presentation2.pdf]
